# Combined effects of acupuncture and auricular acupressure for relieving cancer-related fatigue in patients during lung cancer chemotherapy: A protocol for systematic review and meta-analysis

**DOI:** 10.1097/MD.0000000000027502

**Published:** 2021-10-22

**Authors:** Han Li, Huan Liu

**Affiliations:** a Internal Medicine of Traditional Chinese Medicine, Senior Department of Traditional Chinese Medicine, the Sixth Medical Center of PLA General Hospital, Beijing, China; b Gynecology of Integrated Traditional Chinese and Western Medicine, Beijing Xicheng Guangwai Hospital, Beijing, China.

**Keywords:** acupuncture, auricular acupressure, cancer-related fatigue, lung cancer, meta-analysis, protocol

## Abstract

**Background:**

Increasing attention has been paid to acupuncture and auricular acupressure as alternative strategies for cancer related fatigue (CRF) management. Therefore, we design this systematic review and meta-analysis to explore the efficacy and safety of acupuncture and auricular acupressure for relieving CRF in patients during lung cancer chemotherapy.

**Methods:**

From the inception to August 2021, the Web of Science, EMBASE, PubMed, and Cochrane Library electronic databases were searched using the key phrases “acupuncture”, “auricular acupressure”, and “lung cancer” for all relevant trials. Trials that compared acupuncture (including electroacupuncture) and auricular acupressure with acupuncture alone were included. The primary outcome was the measurement of the CRF symptoms. Secondary outcome measures were physical activity, quality of life, and adverse events. A P value of <.05 was considered to be statistically significant.

**Results:**

It will be the first such study and will obtain evidence for utilizing acupuncture and auricular acupressure for lung cancer patients.

**Conclusion:**

Combined acupuncture and auricular acupressure may be effective for relieving CRF in patients during lung cancer chemotherapy.

## 1 Introduction

Cancer-related fatigue (CRF) is one of the most common subjective unpleasant side effects in patients after chemotherapy, affecting up to 90% of lung cancer patients, and it is a difficult symptom for practicing physicians to manage.^[1]^ CRF is characterized by multidimensional symptoms that affects daily life, social life, family life, work and role functioning, resulting in unpleasant symptoms such as loss of appetite and altered emotional state. However, despite extensive research and ongoing efforts to address the issue, including patient education and physical exercise, practicing clinicians, clinical staff, carers, and patients themselves often view CRF as a corollary of cancer treatment and a symptom that is difficult to treat.^[2,3]^

Currently, there is a lack of accurate and effective drug interventions for the treatment of CRF. Accordingly, complementary and alternative medicine has become increasingly popular among cancer patients and is often used to manage adverse reactions associated with cancer treatment. Traditional Chinese medicine (TCM) holds that the pathological mechanism of CRF mainly includes the lack of vital qi, deficiency of qi and blood, yin and yang, and viscera asthenia, accompanied by an accumulation of phlegm or dampness, stagnation of qi or stasis of blood.^[4–6]^ Consequently, TCM believes that regulating the viscera and tonifying qi and blood are keys to the successful treatment of CRF. In TCM, acupuncture is often used alone or in combination with auricular acupressure to replenish qi and blood.^[7]^ Acupuncture involves inserting fine needles at certain points at specific angles and rotating, lifting, and pricking the needles, based on the theory of TCM. The main therapeutic effects of acupuncture include dredging meridians, regulating Yin and Yang, enhancing body resistance, and eliminating pathogenic factors.^[8–11]^ Auricular acupressure is a branch of traditional Chinese acupuncture. By exerting pressure on acupoints to stimulate meridians, it can affect the release of neurotransmitters transmitting signals along neurons, regulate endocrine and viscera functions, and finally relieve discomfort symptoms and cure some diseases.^[12–14]^

Increasing attention has been paid to acupuncture and auricular acupressure as alternative strategies for CRF management. However, no meta-analysis or review has attempted to analyze the combined effect of acupuncture and auricular acupressure for relieving CRF in patients during lung cancer chemotherapy compared to acupuncture alone. Therefore, we design this systematic review and meta-analysis to explore the efficacy and safety of acupuncture and auricular acupressure for relieving CRF in patients during lung cancer chemotherapy.

## 2 Materials and methods

### 2.1 Search strategy

The Preferred Reporting Items for Systematic Reviews and Meta-Analyses (PRISMA) reporting guidelines will be followed to conduct the present meta-analysis.^[15]^ From the inception to August 2021, the Web of Science, EMBASE, PubMed, and Cochrane Library electronic databases were searched using the key phrases “acupuncture”, “auricular acupressure”, and “lung cancer” for all relevant trials. Moreover, references cited by the relevant sources were also hand-searched to identify any additional articles that could not be found in our database query. Ethical approval and patient consent are not required because this study was conducted based on previous studies. The systematic review protocol has been registered on Open Science Framework registries with registration number 10.17605/OSF.IO/7AB5T.

### 2.2 Eligibility criteria

#### 2.2.1 Participants

Trials that focused on adult lung cancer patients (≥18 years) were included.

#### 2.2.2 Interventions and controls

Trials that compared acupuncture (including electroacupuncture) and auricular acupressure with acupuncture alone were included, and those that focused on laser acupuncture or electroacupuncture without needles were excluded.

#### 2.2.3 Outcomes

Trials with CRF as an outcome were included in a systematic review; among these trials, studies with extractable CRF scores were included in the meta-analysis.

#### 2.2.4 Studies

Cohort studies were eligible.

### 2.3 Data extraction

In order to achieve a consistency (at least 80%) of extracted items, the data extractors extracted data from a sample of eligible studies. Results of the pilot extraction were discussed among review authors and extractors. Two independent reviewers extracted data with a predefined extraction template, which included the following items: study characteristics such as the first author, publication year, study design, follow-up period; patient demographic details such as patients’ number, average age, and gender ratio. The primary outcome was the measurement of the CRF symptoms. Secondary outcome measures were physical activity, quality of life, and adverse events, including nausea, vomiting, anorexia, diarrhea, fever, pain, dizziness, and bleeding. The original authors contacted to request missing data where necessary. Extracted information would be cross-checked by 2 independent reviewers. Any disagreements would be discussed and resolved in discussion with a third reviewer.

### 2.4 Statistical analysis

Review Manager software (v 5.4; Cochrane Collaboration) was used for the meta-analysis. Continuous variables were extracted and analyzed to mean value ± SD. Standardized mean differences with a 95% confidence interval were assessed for continuous outcomes. The heterogeneity was assessed by using the Q test and I^2^ statistic. An I^2^ value of <25% was chosen to represent low heterogeneity and an I^2^ value of >75% to indicate high heterogeneity. All outcomes were pooled on random-effect model. A *P* value of <.05 was considered to be statistically significant.

### 2.5 Quality assessment

The Cochrane risk of bias tool was independently used to evaluate the risk of bias of included randomized controlled studies by 2 reviewers. The quality of randomized controlled studies was assessed by using following 7 items: random sequence generation, allocation concealment, blinding of participants and personnel, blinding of outcome assessment, incomplete outcome data, selective reporting, and other bias. A modified version of the Downs and Black tool was adopted to evaluate the quality of non-randomized cohort studies. The modified version consists of 27 items with a total possible score of 29. A score of ≥75% indicates high quality, 60% to 74% indicates moderate quality, and ≤60% low quality. Two investigators independently evaluated included studies on the 27 criteria, with any discrepancies resolved by a third independent reviewer. Kappa values were used to measure the degree of agreement between the 2 reviewers and were rated as follows: fair, 0.40 to 0.59; good, 0.60 to 0.74; and excellent, 0.75 or more.

## 3 Discussion

Increasing attention has been paid to acupuncture and auricular acupressure as alternative strategies for CRF management. However, no meta-analysis or review has attempted to analyze the combined effect of acupuncture and auricular acupressure for relieving CRF in patients during lung cancer chemotherapy compared to acupuncture alone. This systematic review and meta-analysis aims to evaluate the combined effects of acupuncture and auricular acupressure for relieving CRF in patients during lung cancer chemotherapy. It will be the first such study and will obtain evidence for utilizing acupuncture and auricular acupressure for lung cancer patients. The outcome measures will include CRF symptoms, physical activity, quality of life, and adverse events.

## Author contributions

**Conceptualization:** Han Li.

**Data curation:** Huan Liu.

**Formal analysis:** Huan Liu.

**Funding acquisition:** Han Li.

**Investigation:** Huan Liu.

**Methodology:** Han Li.

**Project administration:** Han Li.

**Software:** Huan Liu.

**Writing – original draft:** Huan Liu.

**Writing – review & editing:** Han Li.
